# Detection and seroprevalence of Q fever infection in dairy goats in Besut district, Malaysia

**DOI:** 10.5455/javar.2024.k768

**Published:** 2024-06-04

**Authors:** Khairunnisa Ahmad, Nik Danial Asyraf Nik Mustaffa, Nur Syafiqa Azmi, Siti Mariam Zainal Ariffin, Mohd Faizal Bin Ghazali, Noor Syaheera Ibrahim

**Affiliations:** 1 School of Animal Science, Aquatic Science and Environment, Faculty of Bioresources and Food Industry, Universiti Sultan Zainal Abidin, Besut, Malaysia.; 2 Department of Veterinary Pre-Clinical Science, Faculty of Veterinary Medicine, Universiti Putra Malaysia, Universiti Putra Malaysia, Serdang, Malaysia.

**Keywords:** Seroprevalence, Q fever, *Coxiella burnetii*, dairy goats, ELISA

## Abstract

**Objective:**

This study aimed to investigate the seroprevalence of Q fever and its association with age and gender among Saanen dairy goats in Malaysia.

**Materials and Methods:**

One hundred dairy goats (*n = *100) aged 6 months to 6 years were randomly selected, and blood samples were collected for serological analysis using the enzyme-linked immunosorbent assay technique.

**Results:**

The results revealed a seropositive rate of 70% among the goats, with medium-positive titers being the most common. The prevalence of Q fever varied among different age groups, with higher rates observed in adult goats aged between 5 and 6 years. Gender analysis showed that males had a higher positive rate (*p* < 0.05) of Q fever compared to females.

**Conclusion:**

These findings strongly indicate the presence of *Coxiella* *burnetii* in the dairy goat population and highlight the importance of implementing biosecurity measures and control strategies to prevent further transmission of this disease. This research has contributed to a better understanding of Q fever epidemiology and provides insights for effective control and prevention strategies in dairy goat populations.

## Introduction

Edward Holbrook Derrick first developed the term “Q fever” in 1937 to describe an inflammatory illness that he saw in workers in slaughterhouses [1]. This infectious condition can affect both human and animal populations; it is categorized as a zoonotic disease. Q fever can present with a wide range of clinical manifestations. These can include nonspecific symptoms such as cough, fatigue, fever, headache, joint pain, and shortness of breath, as well as more severe symptoms that affect specific anatomical regions such as the bone marrow, liver, lungs, spleen, and female reproductive system [2]. The bacterium that causes Q fever is called *Coxiella burnetii* (*C. burnetii*), and it is an intracellular Gram-negative bacterium that is distributed globally. Since *C. burnetii* has been found in both domestic and wild animals, ticks and other arthropods may serve as potential reservoirs [1,3]. Numerous studies have shown that ruminant animals, such as cattle, sheep, and goats, frequently carry *C. burnetii*, indicating their important roles in the bacterium’s survival [3]. Pet animals can act as reservoirs for the pathogen, allowing it to spread to humans and other animals, even though Q fever infections in cats and dogs are rare [3,4]. The urine, feces, and placental membranes of animals that are pregnant or have had an abortion are usually where the causal agent of Q fever is found [5]. As of right now, the most common method for detecting Q fever is still serological analysis, namely using the immunofluorescence assay [6,7].

Q fever presents a significant occupational risk, particularly for workers who have frequent contact with farm and laboratory animals, making them high-risk groups [8]. The disease’s importance for veterinary public health has been highlighted by the numerous notable Q fever outbreaks involving humans and livestock that have been reported in several nations, including the United Kingdom, the Netherlands, the United States, and Hungary, since the disease’s original discovery in Australia [2]. Furthermore, Q fever outbreaks in animal farms—especially dairy farms—present a significant financial risk to farmers because they might cause abortion storms in infected herds, which would reduce the number of available replacement animals and milk supply.

Several investigations conducted in Malaysia have reported that Q fever is common in ruminant farms spread across the Peninsular States [9]. Nevertheless, there is a lack of sufficient evidence concerning the frequency of Q fever and the ecological factors related to *C. burnetii* in dairy goats in Malaysia. Moreover, there are notably few published studies that examine the relationship between pertinent risk factors—such as gender and age—and the severity of Q fever in small ruminants in Malaysia. Therefore, evaluating Q fever in a range of age groups may present a more successful strategy for people as well as the farming sector. Thus, the purpose of this study is to investigate any potential relationships between age and gender variables and the seroprevalence of Q fever antibodies in Saanen dairy goats in Terengganu’s Besut district. We think that by identifying the various age groups and gender variances among Saanen dairy goats, these characteristics would help to improve husbandry practices and offer fresh perspectives for treating and preventing Q fever sickness more thoroughly.

## Materials and Methods

### Authorization for animal ethics

The UniSZA Animal and Plant Research Ethics Committee (UAPREC) of Universiti Sultan Zainal Abidin has approved the research ethics with protocol no. UAPREC/007/031.

### Goat selection procedure

For this investigation, one hundred Saanen dairy goats (*n = *100) were chosen randomly. Following an age-based grouping of the sample population, the distribution was as follows: six months old (*n* = 12), one year old (*n* = 11), two years old (*n* = 20), three years old (*n* = 15), four years old (*n* = 20), five years old (*n* = 2), and six years old and above (*n* = 20). There are 33 males and 67 females in the gender distribution. All dairy goats were kept in an intensive housing system with limited access to grassland, and their body condition ratings ranged from 2.0 to 3.0. In terms of nutrition, the farm staff fed the adult and young goats the same diet based on their body weight each day, which consisted of commercial goat pellets in the morning and freshly cut *Brachiaria humidicola* grass in the afternoon.

### Serum sample collection

A total of 5–8 ml of blood samples were obtained by the jugular vein using a Vacutainer plain tube and stored at a temperature of 4°C in an ice box. According to a previous study [10], the blood naturally divides into serum and cellular components within the same day. For safety purposes, PPE, such as gloves, coveralls, and masks, was used while handling the goats during the sampling procedure.

### ELISA analysis

The serum samples were screened for Q fever-specific antibody testing using the enzyme-linked immunosorbent assay (ELISA). The ELISA test was performed using the manufacturer’s procedure provided by PrioCHECK™ (ThermoFisher Scientific, France), using phase I+II purified antigens obtained from *C. burnetii* strains. The absorbance at 450 nm (monochromatic) was measured using a microplate reader within a maximum time frame of 30 min after the reaction ended. The results were measured as a percentage of the optical density (OD) value. The positive control (PC) showed an OD value of 100%. For serum samples, those with an OD percentage equal to or more than 50% were categorized as ELISA-positive. Specimens with OD values falling within the range of 40% to 50% were deemed uncertain, but those with OD values below 40% were classified as negative.

### Data interpretation and analysis

Phase I and phase II antigens that were obtained from domestic ruminants were used in the indirect ELISA kit. Samples of serum were first diluted 1:400. The S/P ratio (ODsample - ODm PC)/(ODm PC - ODm NC) and Titer = S/P × 100 were used to compute the test results. The classification of serum samples was as follows: negatives for any Titer value below 40, weak positives (++) for any Titer value between 40 and 100, medium positives (++) for any 100 to 200, strong positives (++++) for any 200 to 300, and very high positives (++++) for any Titer value greater than 300.

S/P = (OD sample – ODm_NC_) / (ODm _Pc_ – ODm _NC_)

Titer = S/P × 100

Prevalence = (Number of positive samples) / (Total number of samples)

OD = optical density, PC = positive control, NC = negative control, and S/P = samples/positive ratio.

## Results

### The seroprevalence of Q fever in Saanen dairy goats

As part of this study, 100 goats were tested for Q fever using the ELISA-specific antibody detection technique. Seventy percent (70/100) of the goats tested positive for Q fever, according to the data. Subsequent investigation revealed that 60% (60/100) of the goat samples were in the medium-positive group, representing a sizable part of the overall samples. Additionally, weak positive titers were seen in 10% (10/100) of the samples. On the other hand, 30 out of 100 goat (30%) samples showed negative for Q fever. Interestingly, not a single sample showed high or extremely high positive titers.

**Table 1.** Descriptive analysis of age effect on the titer response of Q fever in Saanen dairy goats.

Age of goats	Mean ± SD		*p* value
	Positive	Negative	
6 months–1-year-old	156.77 ± 13.11	16.58 ± 14.68	**< 0.05**
2 years old	134.5 ± 26.54	14.5 ± 12.47	**< 0.05**
3 years old	231.1	0	NA
4 years old	188.21 ± 100.6	24.5 ± 7.89	**< 0.05**
5 years old	113	0	NA
6 years old	254.1	0	NA

The *p-*value could not be calculated due to an insufficient number of positive or negative samples, as indicated by NA (not applicable).

**Figure 1.**
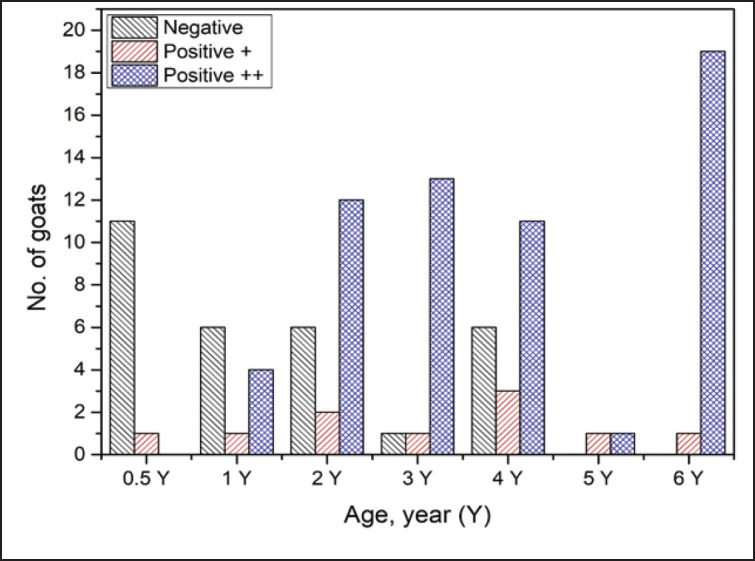
Number of dairy goats infected with Q fever according to age group.

### The impact of age on the prevalence of Q fever in Saanen dairy goats

In this investigation, 91.7% (11/12) of the goats at 6 months of age (0.5 Y) were determined to be negative for Q fever, but all goats between 5 and 6 years were found to be seropositive for *C. burnetii* infection. Meanwhile, the vast majority (95%; 19/20) of goats in the 6-year-old group had moderately positive findings, followed by goats in the 3-year-old group (86.7%; 13/15), as seen in Table 1 and Figure 1. Table 1 presents the data analysis in terms of the mean ± SD. The positive and negative results were examined using a T-test to calculate the *p*-value.

### Gender effect on Q fever infection in Saanen dairy goats

**Figure 2.**
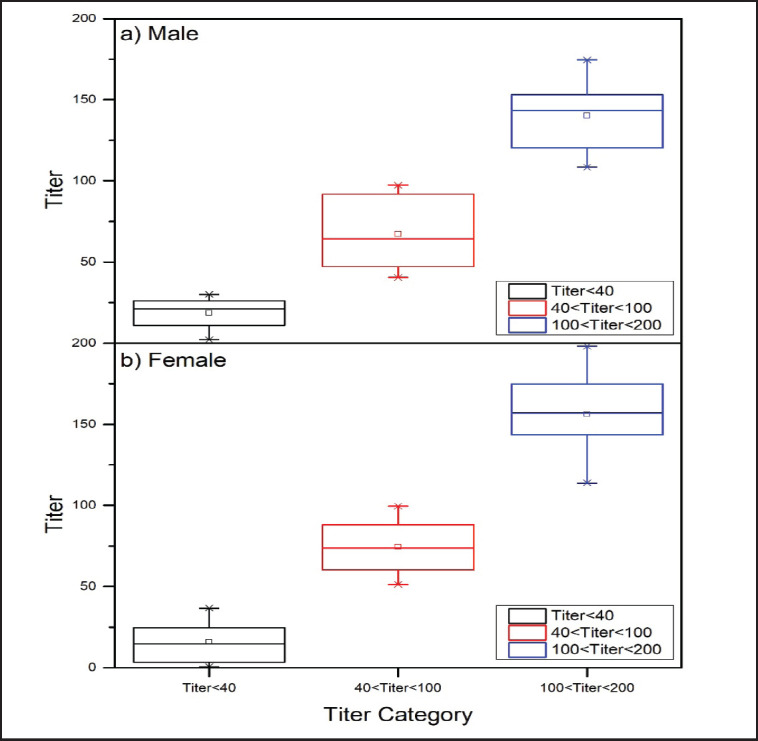
Titer response of Saanen goats with Q fever based on gender classification.

The research findings revealed that 24.2% of the male Saanen goats, specifically 8 out of 33 samples, exhibited a negative result for Q fever upon testing. Out of the 33 male Saanen goat samples, 45.5% (15 out of 33) showed medium positive results for Q fever, while the remaining 30.3% (10 out of 33) fell into the weak positive category. Conversely, the ELISA test showed that 41.8% (28 out of 67) of female Saanen goats tested negative, 19.4% (13 out of 67) had weak positive findings, and 38.8% (26 out of 67) had medium positive results, as shown in Table 2. The statistical analysis demonstrated a notable disparity in the identification of Q fever between genders, as indicated by the findings reported in Table 3. Figure 2 graphically illustrates the titer response of Saanen goats infected with Q fever, considering the categorization based on gender. The reported data offer useful insights into potential changes or discrepancies in the immunological response and antibody production associated with Q fever in Saanen goats, taking into account their gender categorization.

**Table 2.** The seroprevalence result of Q fever in goats according to their genders.

Gender (number of animals)	Negative (%)	Positive (%)			
		+	++	+++	++++
Male (*n = * 33)	8 (24.2)	10 (30.3)	15 (45.5)	0 (0)	0 (0)
Female (*n = * 67)	28 (41.8)	13 (19.4)	26 (38.8)	0 (0)	0 (0)
Total	36 (36.0)	23 (23.0)	41 (41.0)	0 (0)	0 (0)

**Table 3.** Descriptive analysis of gender effect on the titer response of Q fever in Saanen dairy goats.

Gender	Mean		*p* value
	Positive	Negative	
Male (*n = *33)	110.99 ± 42.27	18.55 ± 10.02	*p* < 0.05
Female (*n = *66)	129.66 ± 44.13	15.67 ± 11.89	*p* < 0.05

## Discussion

The presence of Q fever antibodies in livestock animals is a major concern in veterinary public health because of the potential consequences linked to *C. burnetii* infections. The occurrence of these diseases often led to financial losses due to sickness, decreased ability to reproduce, and reduced overall output [11,12]. Regarding goats especially, clinical symptoms of Q fever infection include events such as abortions, stillbirths, and the birth of weak offspring [13,14]. The investigation found that 70% of goats in the Besut District were observed to have Q fever. The value surpasses the observed seroprevalence rate of Q fever in small ruminants from Terengganu and Negeri Sembilan, which is recorded at 12.1% [15]. An independent study conducted in many states in Peninsular Malaysia revealed a somewhat lower occurrence of *C. burnetii*, specifically 12.2%, in biological samples collected from small ruminants [16].

Q fever antibodies detected in a goat herd typically suggest that the goats have been exposed to *C. burnetii* infections in the past or are currently being exposed to them [17]. Most of the goats in this investigation did not show any visible clinical symptoms related to Q fever. This compelling result suggests that these specific animals may have experienced previous episodes of infection, potentially indicating the presence of an immune response or acquired immunity. Prior studies have suggested certain threshold values for diagnosing acute and chronic Q fever through the utilization of the immunofluorescence assay. The authors suggested that a threshold of ≥200 for anti-phase II IgG titers and ≥50 for anti-phase II IgM titers may be used to diagnose acute Q fever. They also suggested that a threshold of ≥800 for anti-phase I IgG titers might be used to diagnose chronic Q fever [27]. Furthermore, Barberio [28] identified weak positive and medium positive titers as markers of mild and moderate Q fever infections. In this study, goats with titers between 100 and 200 were classified as having active Q fever, even if they did not show any obvious indications of *C. burnetii* infections. It is worth mentioning that all the samples that tested positive in this study showed weak or moderate positive levels below 200, which suggests that there were no recent or ongoing Q fever infections. These animals that test positive for antibodies to *C. burnetii* can carry the bacteria without showing any noticeable symptoms or clinical signs [18].

The current study demonstrates that young goats between the ages of 6 months and 1 year exhibit lower infection rates of *C. burnetii*. It is noteworthy that a prior investigation revealed that the age group of animals between 1 and 2 years old saw the greatest impact following the introduction of Q fever into the herd [19]. Additionally, a study conducted in 2017 found that the prevalence of Q fever infection in Saanen goat kids was reduced at 6 months of age compared to one-year-old goats, which aligns with the findings of the current study [13]. However, a prior investigation demonstrated that the majority of Saanen goat kids, previously identified as lacking antibodies, would develop antibodies as they grew older, even in the absence of adult goats. This suggests that the young animals were already contained in or infected with *C. burnetii* before they began giving birth [13]. *Coxiella burnetii* may have inhabited the reproductive organs of young animals and only began to reproduce when the animals’ reproductive organs started to grow, resulting in the later onset of Q fever infection in older animals [20]. Overall, seropositive goats were found in all age categories, indicating that the infections were likely already prevalent and spreading on the farm since the initial arrival of Saanen dairy goats (6 years old and older) some years ago.

The current study is intended to determine the seroprevalence rates of Q fever in both males and females, with a specific focus on identifying the occurrence of positive Q fever cases. Notably, a greater percentage of males (75.8%; 25 out of 33) showed seropositivity for Q fever compared to females (58.2%; 39 out of 67). This result contradicts the results of a prior study [20] that indicated higher rates of infection among females in comparison to males. The pattern remained consistent throughout this analysis, even though gender was not specifically specified as a designated explanatory variable. The variation in outcomes can be attributed to the tendency of *C. burnetii* to mostly invade the female reproductive system, including the uterus and mammary gland. As a result, female goats have a higher likelihood of getting Q fever [21]. Due to the use of random sampling in this study, without considering the animals’ past histories, there is a possibility that more Q fever-positive males were selected. This accounts for the larger proportion of Q fever-affected males compared to females. Notably, the number of female Saanen goats (*n = *26) with a higher degree of infection (medium positive titer) was higher than that of male Saanen goats (*n = *15). This discovery is similar to a prior investigation conducted in France, wherein female goats exhibit more severe and aggressive Q fever infections in comparison to their male counterparts [21]. If variables like a stressful living environment were to be introduced to the does, the quantity of invasive bacteria might grow and the infection would advance to a chronic state [22]. As a result, it is very likely that pregnant female mammals, such as dairy goats, may reactivate their Q fever infection [23,24].

The identification of Q fever seropositivity among Saanen dairy goats in different age and gender groups provides proof of the spread of the disease and the presence of the causative agent at Besut Farm. While the study did not provide conclusive evidence regarding a specific gender or age group, the presence of seropositive cases is noteworthy. This suggests that the transmission of Q fever within the farm may be linked to the management practices and environmental conditions that facilitate the circulation of *C. burnetii* [25,26]. Although the Saanen dairy goats do not show any visible clinical symptoms, there is still a high chance of a Q fever outbreak if relapses were to happen. Hence, it is crucial to enforce strict biosecurity measures, adhere to appropriate sanitation procedures, swiftly remove things that pose a risk, and ensure efficient tick control to prevent any further dissemination of the disease. When animals cannot be rehabilitated, the possibility of culling should be considered.

## Conclusion

The study concluded that the seroprevalence of Q fever among dairy goats in Besut District was found to be 70%. This study yielded significant findings about the frequency and factors that contribute to the occurrence of Q fever in small ruminants in Malaysia. The findings demonstrated that age and gender exerted a substantial influence on the prevalence of Q fever infection. Goats between 6 months and 1 year old had reduced rates of infection, whereas older goats, especially those aged 5 and 6 years, had greater levels of seropositivity. Male individuals demonstrated a greater occurrence of Q fever in comparison to females, possibly because the bacterium has a tendency to invade the female reproductive system. The study emphasizes the significance of implementing biosecurity measures, immunization, tick control, and sanitation practices, which are crucial for controlling the spread of Q fever. Biosecurity measures can include practices such as controlling animal movement, restricting access to infected areas, and proper disposal of animal waste. Immunization helps build immunity against the disease, especially in high-risk populations such as farmers and veterinarians. Tick control is essential because ticks can serve as vectors for the bacteria that cause Q fever. Good sanitation practices help in preventing the transmission of bacteria from contaminated environments to humans. By implementing these measures, we can effectively reduce the spread of Q fever and protect both human and animal health. By implementing these proactive measures, dairy farms can effectively mitigate the danger of Q fever transmission and safeguard the well-being of both livestock and farm personnel. This study has enhanced our comprehension of Q fever epidemiology and offers valuable insights for implementing efficient control and preventative measures in dairy goat herds.

## List of Abbreviations

ELISA, enzyme-linked immunosorbent assay; Ig, Immunoglobulin; NC, negative control; OD, optical density; PC, positive control; PPE, personal protective equipement; Q fever, query fever; S/P, samples/positive ratio; UAPREC, UniSZA Animal and Plant Research Ethics Committee; Y, year.
